# Changes in freezing rain occurrence over eastern Canada using convection-permitting climate simulations

**DOI:** 10.1007/s00382-022-06370-6

**Published:** 2022-07-02

**Authors:** Sébastien Marinier, Julie M. Thériault, Kyoko Ikeda

**Affiliations:** 1 Centre ESCER, Department of Earth and Atmospheric Sciences, Université du Québec à Montréal, Montréal, Québec Canada; 2 NCAR National Center for Atmospheric Research, P. O. Box 3000, 80307‑3000 Boulder, Colorado USA

**Keywords:** Freezing rain, Convection-permitting, Winter storms, Global warming

## Abstract

Freezing precipitation has major consequences for ground and air transportation, the health of citizens, and power networks. Previous studies using coarse resolution climate models have shown a northward migration of freezing rain in the future. Increased model resolution can better define local topography leading to improved representation of conditions that are favorable for freezing rain. The goal of this study is to examine the climatology and characteristics of future freezing rain events using very-high resolution climate simulations. Historical and pseudo-global warming simulations with a 4-km horizontal grid length were used and compared with available observations. Simulations revealed a northerly shift of freezing rain occurrence, and an increase in the winter. Freezing rain was still shown to occur in the Saint-Lawrence River Valley in a warmer climate, primarily due to stronger wind channeling. Up to 50% of the future freezing rain events also occurred in present day climate within 12 h of each other. In northern Maine, they are typically shorter than 6 h in current climate and longer than 6 h in warmer conditions due to the onset of precipitation during low-pressure systems occurrences. The occurrence of freezing rain also locally increases slightly north of Québec City in a warmer climate because of freezing rain that is produced by warm rain processes. Overall, the study shows that high-resolution regional climate simulations are needed to study freezing rain events in warmer climate conditions, because high horizontal resolutions better define small-scale topographic features and local physical mechanisms that have an influence on these events.

## Introduction

When surface temperatures are near 0 °C, precipitation in winter storms can take the form of snow, freezing rain, or ice pellets, or there can be a mixture of precipitation types. The accumulation of ice on surfaces can cause major power outages (Bendel and Paton 1981; Regan 1998), ground and air transport disruptions (Martner et al. 1992; Rauber et al. 1994), and injury (Lecomte et al. 1998). These types of events are very common in Eastern Canada (Cortinas et al. 2004) and in particular, within the Saint-Lawrence River Valley (SLRV). The low-level north-easterly winds of the SLRV (Carrera et al. 2009) produce favorable conditions for freezing rain (Ressler et al. 2012).

Freezing rain and ice pellets are usually formed when there is a melting layer above a sub-freezing layer (e.g., Stewart 1992). Ice particles that completely melt may become supercooled before reaching the surface and become freezing rain (Stewart et al. 1990; Zerr 1997; Thériault et al. 2006). If partial melting occurs or if the droplets refreeze within the refreezing layer, the hydrometeors reaching the surface are ice pellets. It is also possible to observe a mixture of precipitation types due to the size distribution of ice particles. The sublimation process in the melting layer aloft can increase the occurrence of ice pellets by decreasing the melting rate (Hanesiak and Stewart 1995). In contrast, freezing precipitation can also be formed through a warm rain process, which does not involve any ice phase precipitation (Huffman and Norman 1988). This process is commonly observed in the Arctic (Roberts and Stewart 2008), the United States (Bernstein 2000; Rauber et al. 2000), and in Canada (Kochtubajda et al. 2017). Although this process is generally linked to freezing drizzle, some observations of freezing rain without a melting layer aloft have been reported.

Warming does not occur evenly across areas affected by changes in climate (IPCC 2013). Therefore, the timing and location of freezing rain events are expected to change in the future. Lambert and Hansen (2011) used a precipitation typing algorithm with a general circulation model (GCM) and found that the maximum of freezing rain events will be shifted poleward for eastern Canada. They also found an overall decrease in future freezing rain event frequency. However, Cheng et al. (2011) used statistical downscaling and a precipitation typing algorithm with GCM simulations and found an overall increase in freezing events. They found that the number of events decrease in October, November, and April, but increase during the winter. Finally, Matte et al. (2019) showed a decrease in freezing rain occurrence frequency over southern Québec in the future. They determined this using the Fifth generation of the Canadian Regional Climate Model (CRCM5) and multiple diagnostic algorithms to diagnose precipitation type. They demonstrated that higher resolution model simulations improve the representation of the topography that is responsible for cold layer formation near the surface. Additionally, higher resolution models, such as convection-permitting climate models (CPCM), explicitly solve the cloud and precipitation processes by using a bulk microphysics scheme (e.g., Thompson and Eidhammer 2014), whereas coarser resolution RCMs generally use empirically-based cloud and precipitation schemes (Sundqvist et al. 1989), even though prognostic precipitation is implemented in some of these models (Gettelman and Morrison 2015; Song et al. 2012).

Freezing precipitation can be destructive and costly. Therefore, it is critical to improve our knowledge on where and when freezing rain events will occur under changing climate conditions. The goal of our study is to investigate how the duration, location and atmospheric conditions that lead to freezing rain events over eastern Canada will change in the future. To achieve this goal, we analyzed 4-km Weather Research and Forecasting (WRF) simulations for current and future climates using the Pseudo Global Warming (PGW) approach (Liu et al. 2017). The simulations available were from 2000 to 2013. First, we compared the climatology of freezing rain with available observations from the NOAA Integrated Surface Database (ISD) (Smith et al. 2011). Second, we investigated the atmospheric conditions associated with the occurrence of freezing rain and how they change in a future climate scenario. These conditions include the vertical temperature structures as well as the surface wind speed and direction.

This paper is organized as follows. Section 2 is a description of the methodology, including the simulation details, the available databases, and data analysis. Section 3 describes the climatology of freezing rain and related variables over the domain of interest. Section 4 covers the climatology of freezing rain events. Section 5 discusses local changes in conditions linked with freezing rain occurrences. Finally, Sect. 6 provides a summary and the conclusions of our study.

## Datasets and methodology

### Description of the WRF simulations

**Fig. 1:**
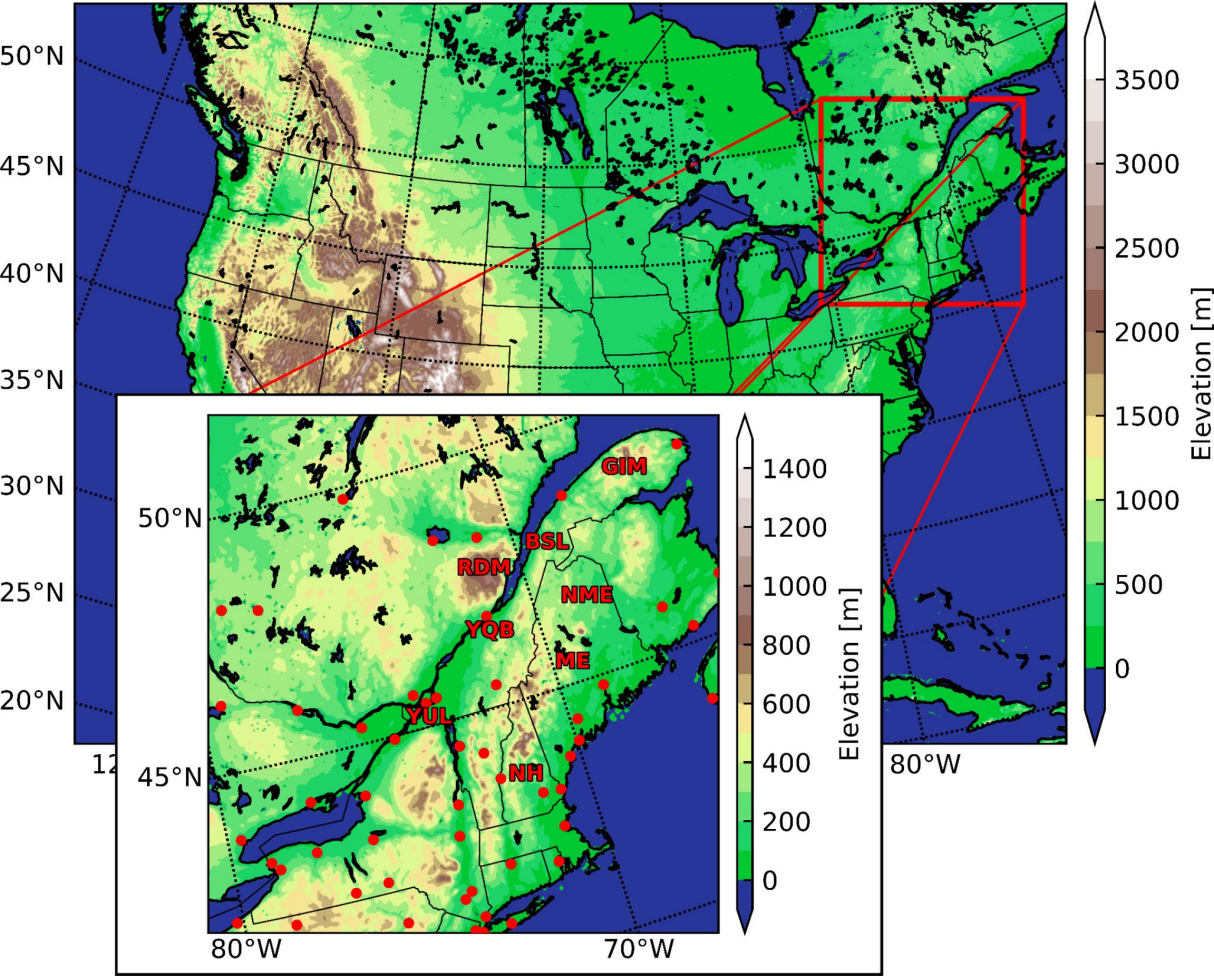
Domain of the simulations and domain of interest (inset) as well as the locations of the NOAA Integrated Surface Database (ISD) stations (red dots) and the locations of interest for this study

High-resolution (4-km horizontal grid spacing) current and future regional climate WRF simulations over the continental U.S. (Rasmussen and Liu 2017; Liu et al. 2017) were used in this study. The domain of the simulations is shown in Fig. 1. The Thompson aerosol-aware microphysics scheme (Thompson and Eidhammer 2014) was used to simulate precipitation types. No parameterised convection schemes were used, as convection is explicitly resolved by the model dynamics and microphysics. Spectral nudging was used above the planetary boundary layer for waves longer than 2000 km. The output frequency is hourly for the 2-D horizontal fields and every 3 h for the 3-D fields.

The future climate simulation was achieved using a PGW approach (Schär et al. 1996; Sato et al. 2007; Hara et al. 2008; Kawase et al. 2009; Rasmussen et al. 2011, 2014). Changes in the physical fields are determined with monthly means over two 30-year periods from the 95-year CMIP5 multi-model ensemble using the RCP8.5 emission scenario. The historical period was 1976–2005 and the future one was 2071–2100. Then the monthly mean signal changes are added to the ERA-Interim reanalysis (0.7° grid-spacing) at initialization and every 6 h at the model boundary, to drive the model. The perturbed variables are geopotential height, specific humidity, horizontal wind, temperature, sea surface temperature, soil temperature, sea level pressure, and sea ice. Further information on the details of the simulations and the PGW experiment can be found in Liu et al. (2017).

The PGW approach can be used to assess the thermodynamic response of warmer and wetter atmospheric conditions. This method allows for the representation of realistic atmospheric circulation in the historical climate, since the initiation of the synoptic systems at the lateral boundaries is coherent with the re-analysis in both simulations. The main limitation of the PGW approach is the assumption that the atmospheric circulation does not change at the lateral boundaries of the domain. Some studies using different approaches report conflicting results about future changes in atmospheric circulation (James 2006; Salathé et al. 2008; Shepherd 2014; Michaelis et al. 2017). The PGW simulations can be applied to a global warming scenario, provided one assumes that similar weather systems occur in similar conditions.

An advantage of the CPCM is that it provides a better representation of topography that leads to accurately reproduced orographic effects on precipitation (Ban et al. 2014; Kendon et al. 2012; Prein et al. 2013; Lind et al. 2020). Additionally, compared to models with coarser horizontal and vertical resolutions, small-scale frontal systems should be better reproduced by these models, since they explicitly predict clouds and precipitation (Oertel et al. 2019; Lind et al. 2020). This can, in turn, improve large-scale representations of synoptic conditions (Schaaf and Feser 2018). Therefore, these simulations are appropriate for studying freezing rain, as many of the events near the Appalachians, the Great Lakes, and the SLRV are driven by complex local topography (Forbes et al. 1987; Stauffer and Warner 1987; Bell and Bosart 1988; Fritsch et al. 1992; Cortinas 2000; Bailey et al. 2003; Kristovich and Spinar 2005; Carrera et al. 2009).

### Available observation databases

The simulations were evaluated using available databases. Total accumulated precipitation was compared to the Canadian Precipitation Analysis (CaPA). CaPA is a 10-km gridded, 6-h precipitation analysis that uses the Regional Deterministic Prediction System from Environment and Climate Change Canada (ECCC) combined with observations from METAR, SYNOP observations (from the Global Telecommunication System), reports from the Réseau Météorologique Coopératif du Québec, and reports from the Standard Hydrometeorological Exchange Format (Mahfouf et al. 2007). In addition, observations from the National Oceanic and Atmospheric Administration Integrated Surface Database (NOAA ISD) were used to compare hourly occurrences of freezing rain at 52 surface stations (McCray et al. 2019). The locations of the surface sites are shown in the inset in Fig. 1. Because the amount of freezing rain was not reported in the observations, only the occurrence of freezing rain is considered for the evaluation and mainly used for the analysis.

### Data analysis methodology

The Thompson microphysics scheme produces surface rain, snow, and graupel fields, making it necessary to diagnose freezing rain. To compare simulated freezing rain with observations, freezing rain is diagnosed in the model when liquid precipitation is produced at the surface and the 2-m temperature is lower than 0 °C. The occurrence of freezing rain is diagnosed offline from the hourly outputs. An hour of freezing rain is only counted when the hourly accumulated liquid precipitation is at least 0.2 mm. This threshold was chosen based on the ECCC definition of trace amount (ECCC 2021), which is the minimum measured amount of precipitation required to be counted as a day of precipitation. This value brings the freezing rain occurrence frequencies closer to the observed values (not shown), as opposed to the 1 mm/day threshold that was used in some previous studies Lambert and Hansen 2011; Cheng et al. 2011; Matte et al. 2018; St-Pierre et al. 2019). The 0.2 mm/h threshold was also used to identify significant precipitation transition regions (Almonte and Stewart 2019).

The duration of freezing rain events in the simulations is defined as the number of hours during which freezing rain occurs. Occurrences are considered to be separate events if more than 6 h elapsed between them. For example, two 1-h occurrences within a 4-h timespan are considered to be a single, 2-hour-long event. Because similar synoptic systems are produced by the PGW method, freezing rain events are defined as concurrent when an event occurs in the PGW simulation within 12 h after the end or before the beginning of an event in the control simulation (CTRL). It is assumed that these events are linked to similar synoptic conditions.

Three-dimensional analyses were conducted at four locations: two in the SLRV in Montréal (YUL) and Québec City (YQB), one in a Rivière-du-Moulin wind farm (RDM), and one in northern Maine (NME). Significant changes in freezing rain occurrences between the CTRL and PGW simulations occurred at RDM and NME. Since 3-D variables were only available at 3-h intervals, only freezing rain that occurred during times that are multiples of three were used to compute quantities linked with 3-D variables. These quantities include geopotential height, wind and temperature at specific pressure levels, sea-level pressure, and melting/refreezing layer thickness and height. The number of occurrences from 3-hour data was confirmed to be nearly a third of the hourly occurrences for both simulations at all locations. This means that proportionally, the 3-hour analyses can be paired with the hourly analyses for the 2-D fields.

## Comparison between the current climate simulation and observations

**Fig. 2:**
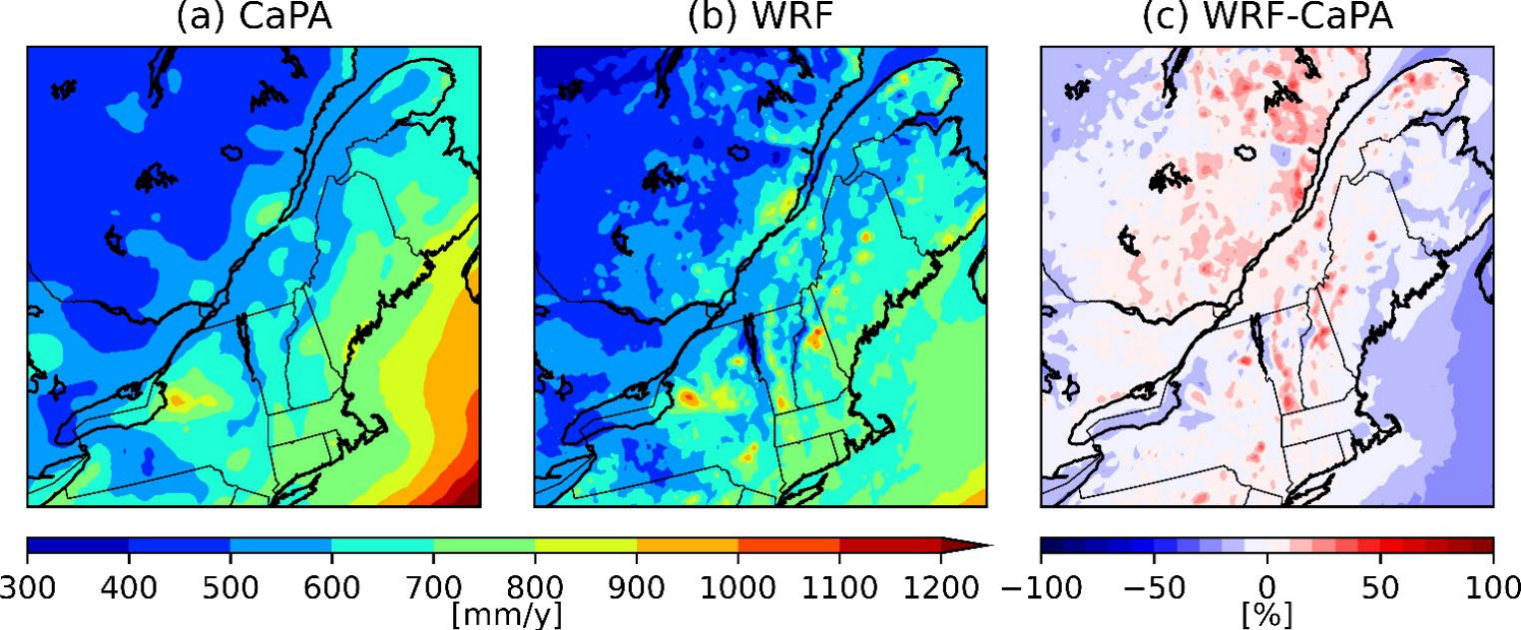
Total accumulated precipitation (October to April) from 2002 to 2012 for (a) CaPA, (b) WRF and (c) the difference (in %) between WRF and CaPA. The WRF data was re-gridded to the 10-km grid of CaPA observations

**Fig. 3:**
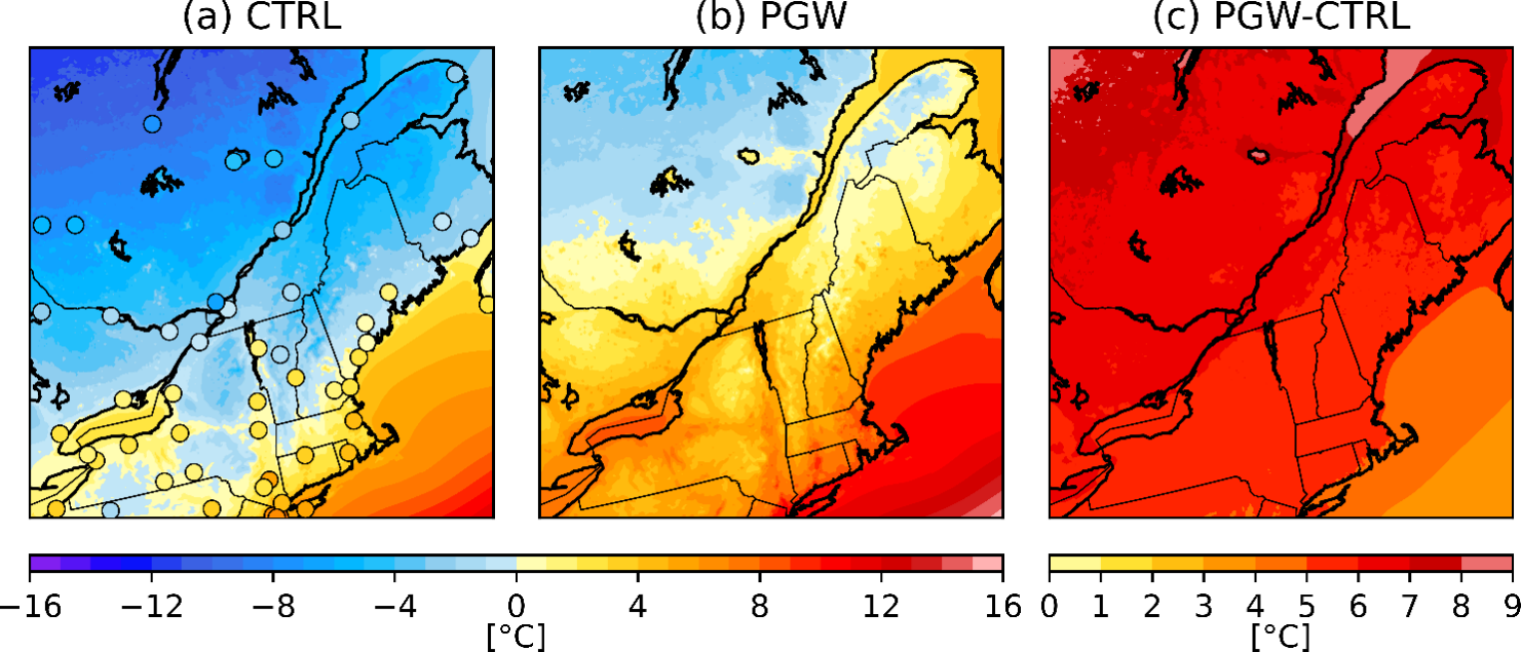
Mean 2-m temperatures from October to April for (a) current and (b) future climate, and (c) the difference between the two climates. The circles represent observations from the NOAA ISD stations

To evaluate the total precipitation fields produced, the WRF model was re-gridded on the 10-km grid of the CaPA product and compared with these between October and April from 2002 to 2012 (Fig. 2). The total accumulated precipitation was similar in both CaPA and the historical WRF simulation, both showing local maxima at the same locations, near YQB, in New Hampshire (NH) and in NME. There was an overestimation of up to 40% over the northeastern part of Quebec, as well as over mountainous regions of Vermont (VT) and New York (NY). The overestimation could be because very few solid precipitation measurements are assimilated in the CaPA system (Lespinas et al. 2015; Schirmer and Jamieson 2015). The mean temperature in the model for the October–April period is about 1 to 3 °C colder than the observations near bodies of water and over the northern part of the domain (Fig. 3a).

**Fig. 4:**
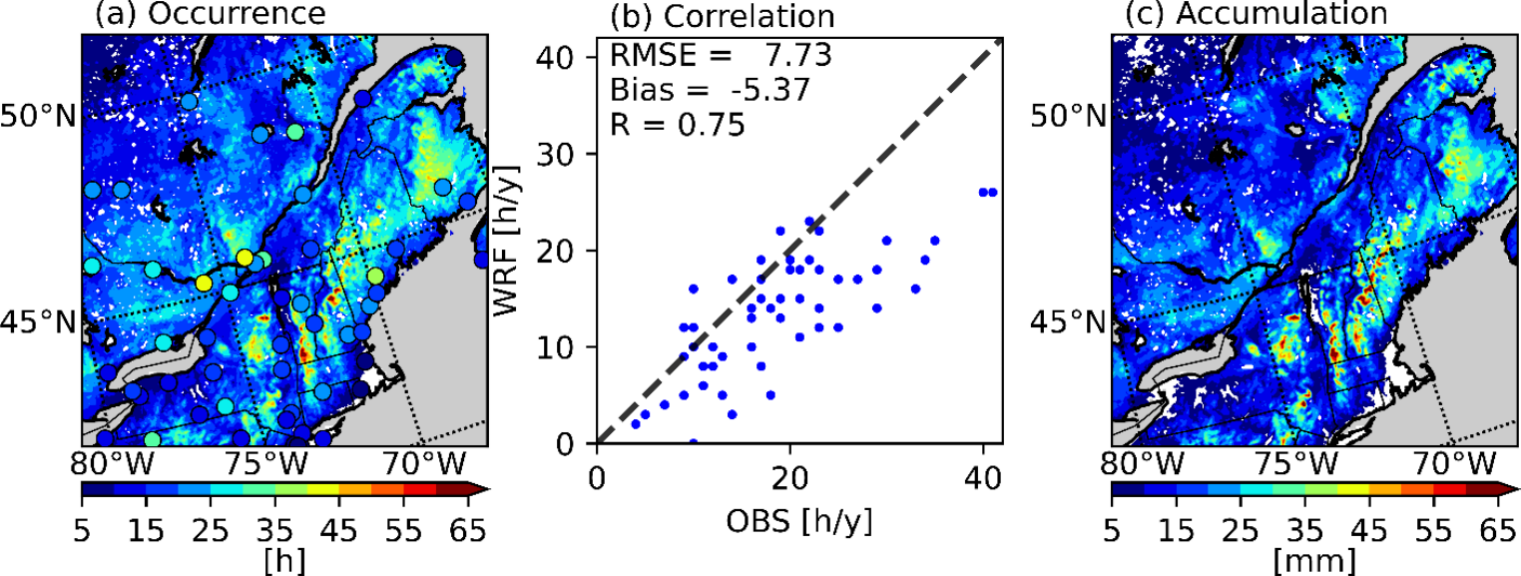
(a) Median annual hours of freezing rain from 2000 to 2013 over the domain of interest, compared with the NOAA ISD (circles); (b) comparison between the median annual hours of freezing rain for the model and observations (OBS) with the dashed line showing the 1:1 slope; (c) median annual accumulation of freezing rain

**Fig. 5:**
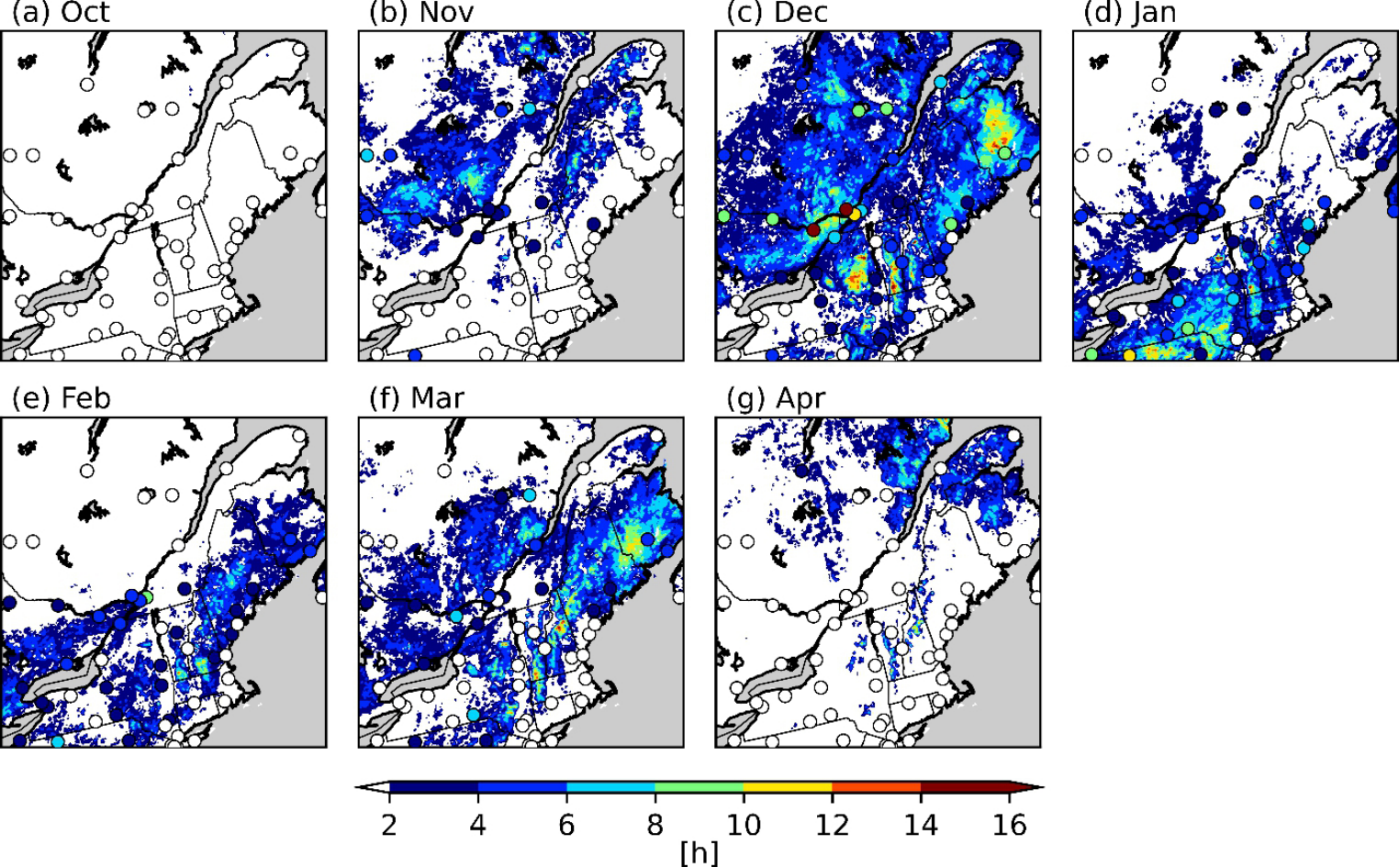
Median annual hours of freezing rain from 2000 to 2013 for the months of October to April in the CTRL simulation, compared with the observations from NOAA ISD (circles) for the same period

The median annual number of hours and accumulation of freezing rain from the current climate simulation are shown in Fig. 4. At most stations, the reported number of hours of freezing rain (R = 0.75) was successfully reproduced by the model. This number was underestimated at some stations, particularly those where more hours of annual freezing rain were observed. This could be in part due to the minimum precipitation threshold of 0.2 mm/h that was used to compute the freezing rain occurrence frequency. Over the studied domain, the patterns of annual freezing rain accumulation were similar to the patterns of freezing rain occurrences. This was expected, since the mean precipitation rate for freezing rain is 0.83 mm/h and the standard deviation is only 0.19 mm/h. Therefore, heavy freezing rainfall was extremely rare and the number of hours follows closely with the total accumulation, in terms of horizontal distribution. This means that accumulated freezing rain at the surface during precipitation events can be linked to its duration. This was also found in Cortinas (2000), Ressler et al. (2012) and McCray et al. (2020). In the latter, they also found that the accretion is closely linked to the duration of a freezing rain event. Finally, at YUL, the monthly occurrence of freezing rain (Fig. 5) is well-reproduced for November and January and underestimated in December and February. In contrast, the model produced freezing rain in April in the northern coastal regions, while no freezing rain was observed at the stations in this region at this time.

## Predicted changes in future climate

### Changes in the occurrence of freezing rain

**Fig. 6:**
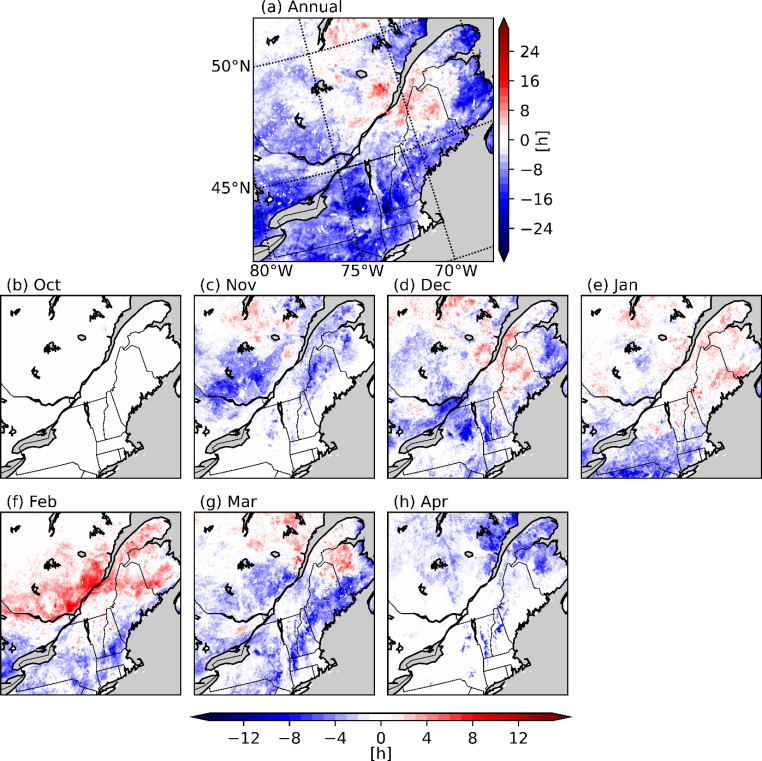
Changes in median (a) annual hours of freezing rain between CTRL and PGW, and (b)–(h) for each month from October to April

Precipitation and 2-m temperatures are expected to change in warmer climate conditions (Figs. 3 and 6) of the PGW simulation used in this study. Since previous studies (Cheng et al. 2011; Klima and Morgan 2015) predicted a poleward shift and more freezing rain occurrences during the winter, we investigated the changes in freezing rain median annual hours both yearly and for each month (Fig. 6). The PGW model showed that there is a decrease of 6 to 32 h/y in the median annual hours of freezing rain over southern Québec and the northeastern United States. In contrast, there is a slight increase (2 to 12 h/y) in the northern part of Québec and in the SLRV between Montréal and Gaspésie-Îles-de-la-Madeleine (GIM). As expected, there is an overall decrease in November, March, and April (up to -10 h/y), and an increase in freezing rain in February (up to 12 h/y), especially north of 45°N. Furthermore, the mean temperature is closer to 0 °C in the PGW simulation compared to CTRL in areas associated with an increase in freezing rain occurrences in the warmer climate scenario.

### Changes in freezing rain events

**Fig. 7:**
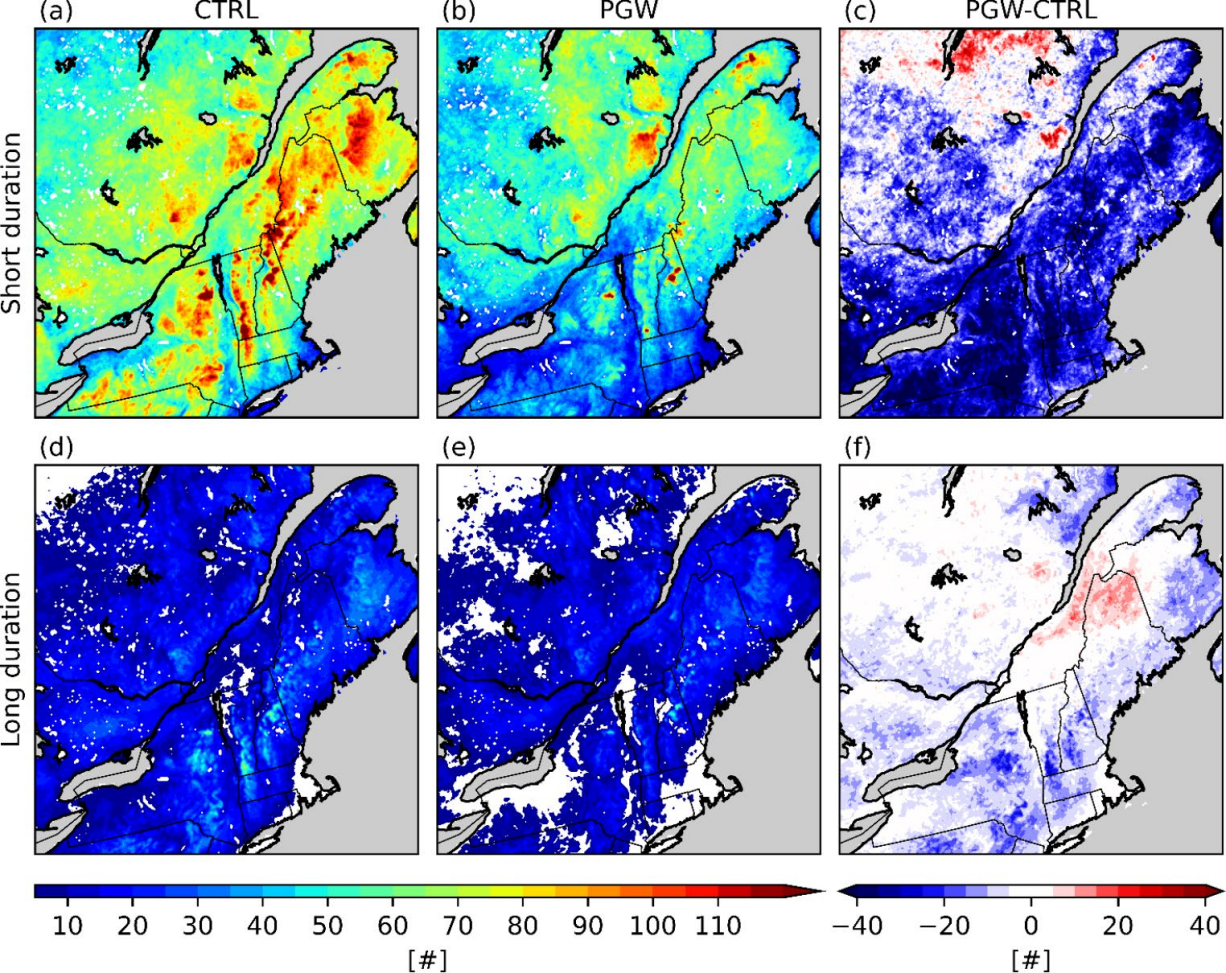
Number of (a)-(c) short-duration (< 6 h) and (d)-(f) long-duration (> 6 h) events over the domain of interest during the 13-year simulation

**Fig. 8:**
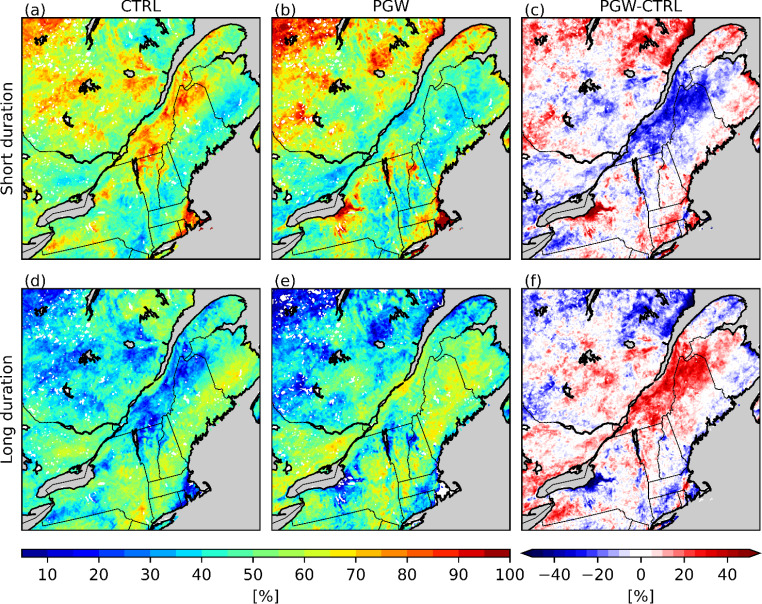
Contribution (%) of (a)–(c) short-duration and (d)–(f) long-duration events to the total freezing rain hours

The spatial distribution of short- and long-duration events (Fig. 7) and their contribution to the total freezing rain occurrences (Fig. 8) were investigated to better understand changes in the climatology of freezing rain. The increase in freezing rain occurrence over Bas-Saint-Laurent (BSL; Fig. 1) in the PGW simulation can be mainly attributed to there being two times as many long-duration events as in the CTRL simulation, which accounts for 15–20% of the total freezing rain hours. The short-duration events are less frequent (15 to 25 events) and correspond to a 5–10% decrease in the total freezing rain hours over this area. At the Rivière-du-Moulin wind farm (RDM) in western Charlevoix, there is an increase from 20 to 30 short-duration events and from 5 to 10 long-duration events. South of the SLRV and near the Great Lakes, there is a general decrease of up to 30 short-duration events. Finally, a slight decrease of the number of short-duration events and a slight increase of the number of long-duration events are observed over Montréal (YUL), which can be explained by the changes in the wind channelling effects in the SLRV (Sect. 5.2).

### Concurrent freezing rain events

**Fig. 9:**
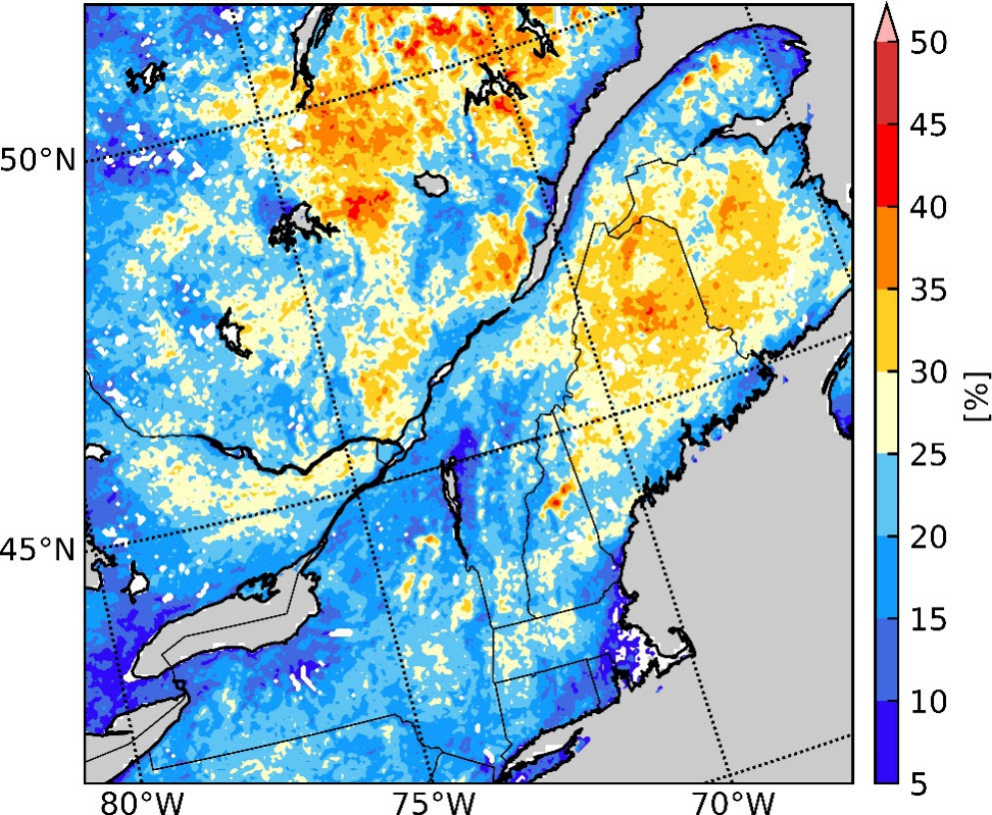
Percentage of freezing rain events in the current climate that also occurred in the warmer climate within 12 h (concurrent events)

**Fig. 10:**
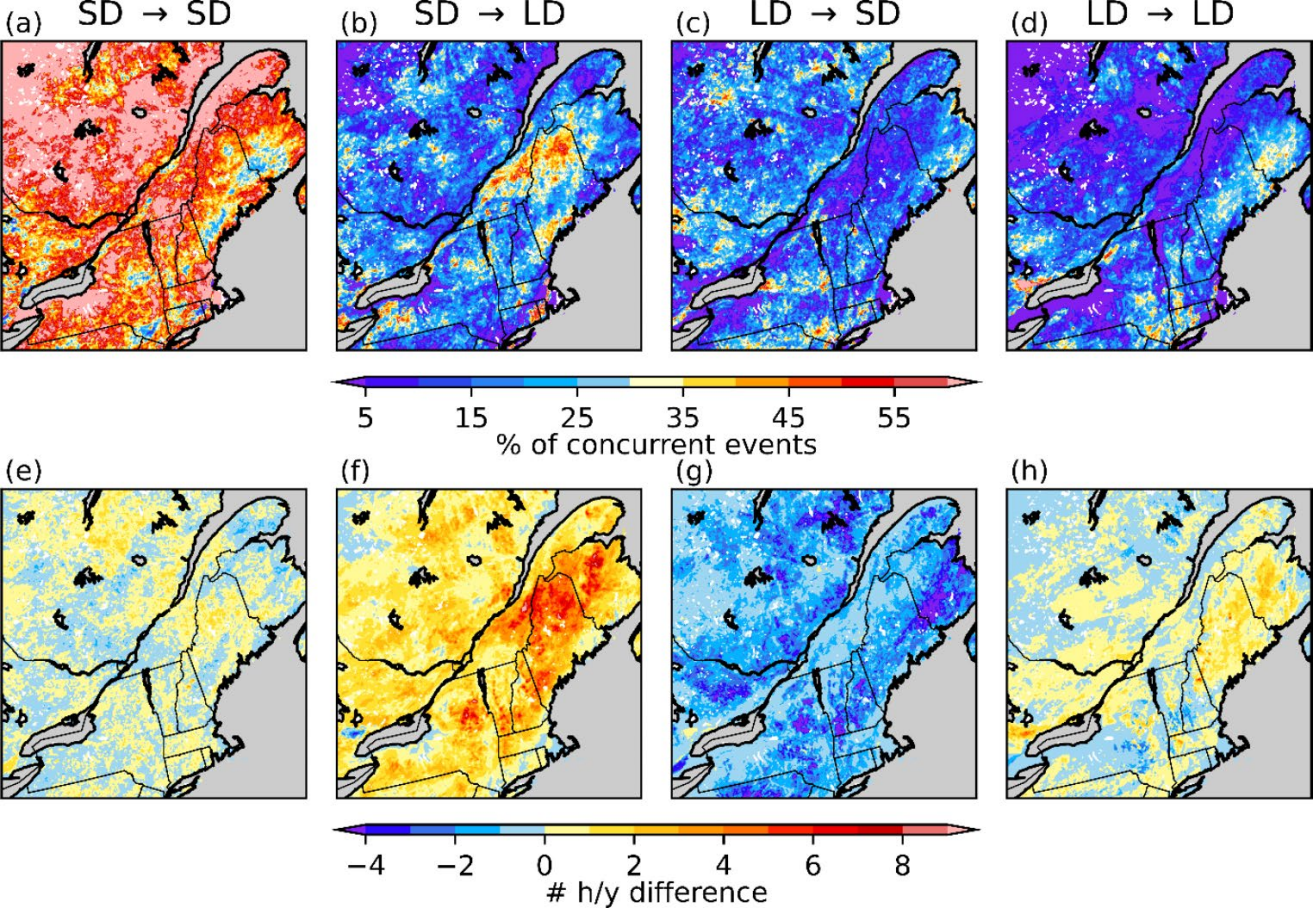
(a)–(d) Percentage of concurrent events that change from short (SD) or long (LD) durations in the CTRL simulation to SD or LD in the PGW simulation. (e)–(h) Number of freezing rain hours associated with these changes

Since the storm tracks and synoptic conditions are nearly identical for freezing rain events that occur in both simulations at approximately the same time (concurrent events), the changes in duration that occur between CTRL and PGW simulations are mainly due to thermodynamic factors. Up to 60% of the freezing rain events in the current climate are concurrent with PGW events (Fig. 9). The maxima of concurrent events (30–60%) are generally located where there are higher occurrences of freezing rain in the PGW simulation, meaning the freezing rain events that occur in the CTRL simulation also occur within 12 h in the PGW simulation. Hence, many of the freezing rain events are associated with the same initial synoptic-scale forcing in both simulations. Among these concurrent events, less than 10% remained the same or decreased in duration (Fig. 10). Up to 55% increased in duration in the PGW simulation over BSL and NME. For this region, the transition from long-duration events (CTRL) to short-duration events (PGW) accounts for a decrease of less than 2 h per year, while the transition from short-duration (CTRL) to long-duration (PGW) events represent an increase of 5 to 9 h/y. Therefore, the events in this region that evolve from short-duration (CTRL) to long-duration (PGW) are responsible for most of the total increase in freezing rain (6 to 12 h/y).

## Changes in local conditions

### Events in the Saint-Lawrence River Valley

**Fig. 11:**
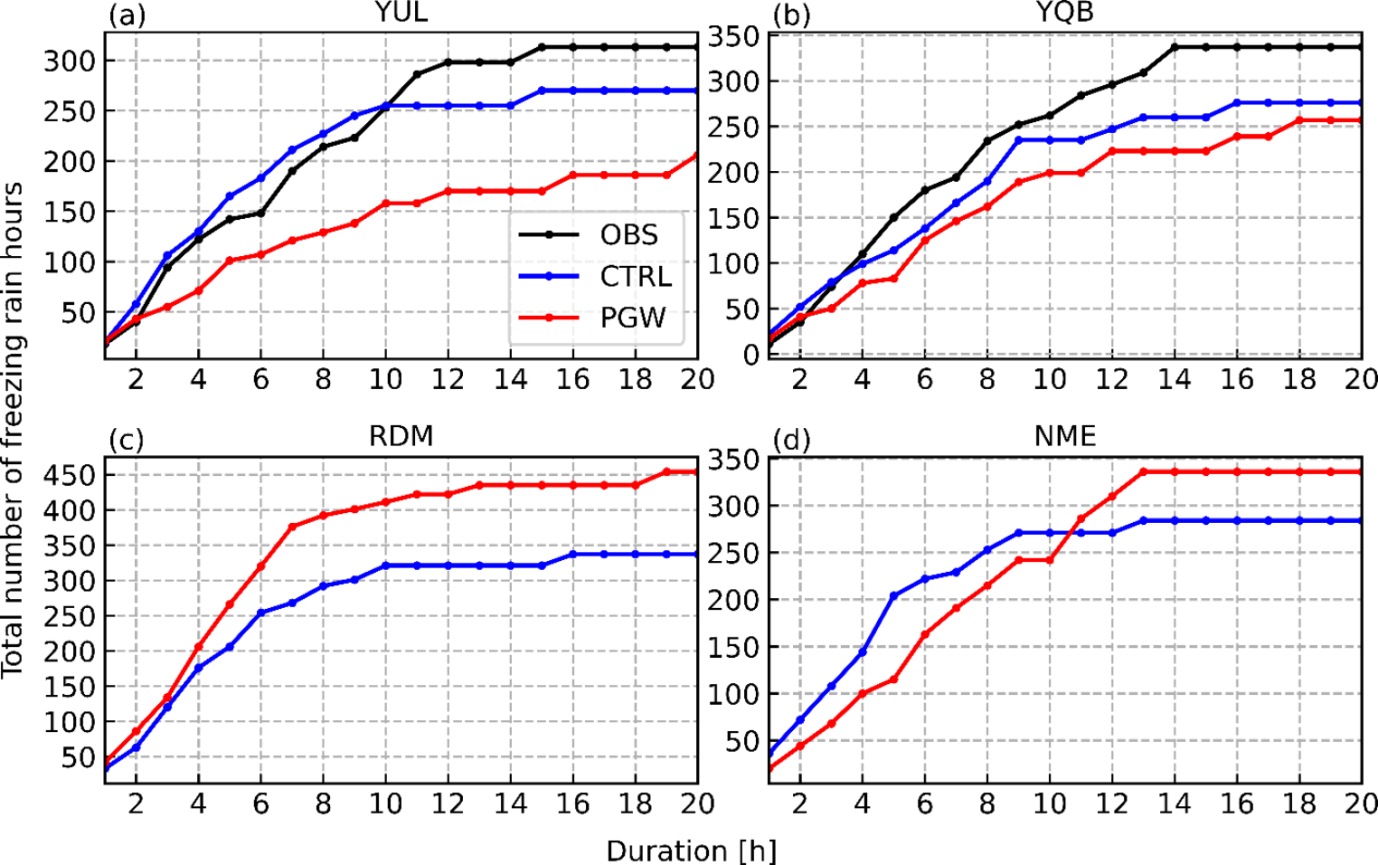
Cumulative distributions of freezing rain events weighted by duration at (a) YUL, (b) YQB, (c) RDM and (d) NME for both simulations and observations (YUL and YQB only)

Even though the warmer climate in the PGW simulation is associated with warmer surface temperatures (2–4 °C), some areas show only small changes in annual freezing rain occurrence (Fig. 6a). This is the case for YUL, where there is a 1-h increase in median annual freezing rain occurrence from CTRL to the PGW simulation (18 and 19 h, respectively). Freezing rain events in both simulations and observations were evaluated for stations in the SLRV (Fig. 11; YUL and YQB). At both locations, the number of short-duration events tends to be overestimated in the historical simulation compared to the observations, whereas the number of long-duration events is slightly underestimated (Fig. 11a, b). This indicates that simulated freezing rain events are generally shorter than the observed events, leading to the underestimation of freezing rain occurrence frequencies at these locations. At YUL, the median number of annual freezing rain hours is 23 h for the observations and 18 h for CTRL. The median is similar (19 h) in the PGW simulation, despite warmer conditions. Despite similar median values between both simulations, the majority of freezing rain hours in the current climate are during events lasting < 9 h, whereas more events have a duration ≥ 9 h in the future climate. At YQB, the simulated (CTRL) median (22 h) is close to the observed median (23 h). There is a slight increase to 25 h in the PGW simulation, compared to the CTRL simulation.

### Physical mechanisms in the Saint-Lawrence River Valley

**Fig. 12:**
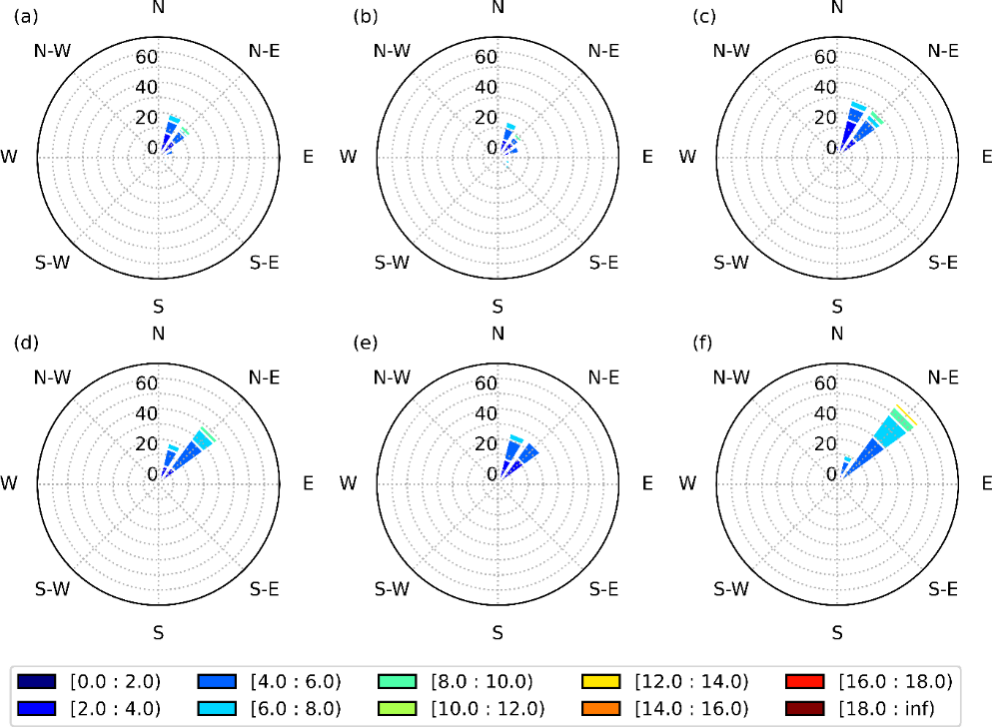
Wind speed and direction distributions (in %) for (a)-(c) CTRL and (d)-(f) PGW simulations during (a, d) all, (b, e) SD and (c, f) LD freezing rain events at YUL. The colormap represents the wind speed (m/s) distribution

Wind channelling in the SLRV contributes to the subfreezing surface temperatures, even in warmer conditions. Near-surface (10-m) wind distributions were generated at YUL (Fig. 12) in the SLRV. It was found that northeasterly winds are dominant during freezing rain events in both simulations due to wind channeling in the SLRV, especially for long duration events. However, they are slightly more frequent in the PGW simulation (82.3%) than in the current climate (69.5%). In the warmer climate, wind speed is higher during long-duration freezing rain events. Similar conditions occur at YQB. In summary, stronger northeasterly winds enhance low-level cold-air advection and could explain the small change in the occurrence of annual freezing rain in warmer conditions in the valley.

**Fig. 13:**
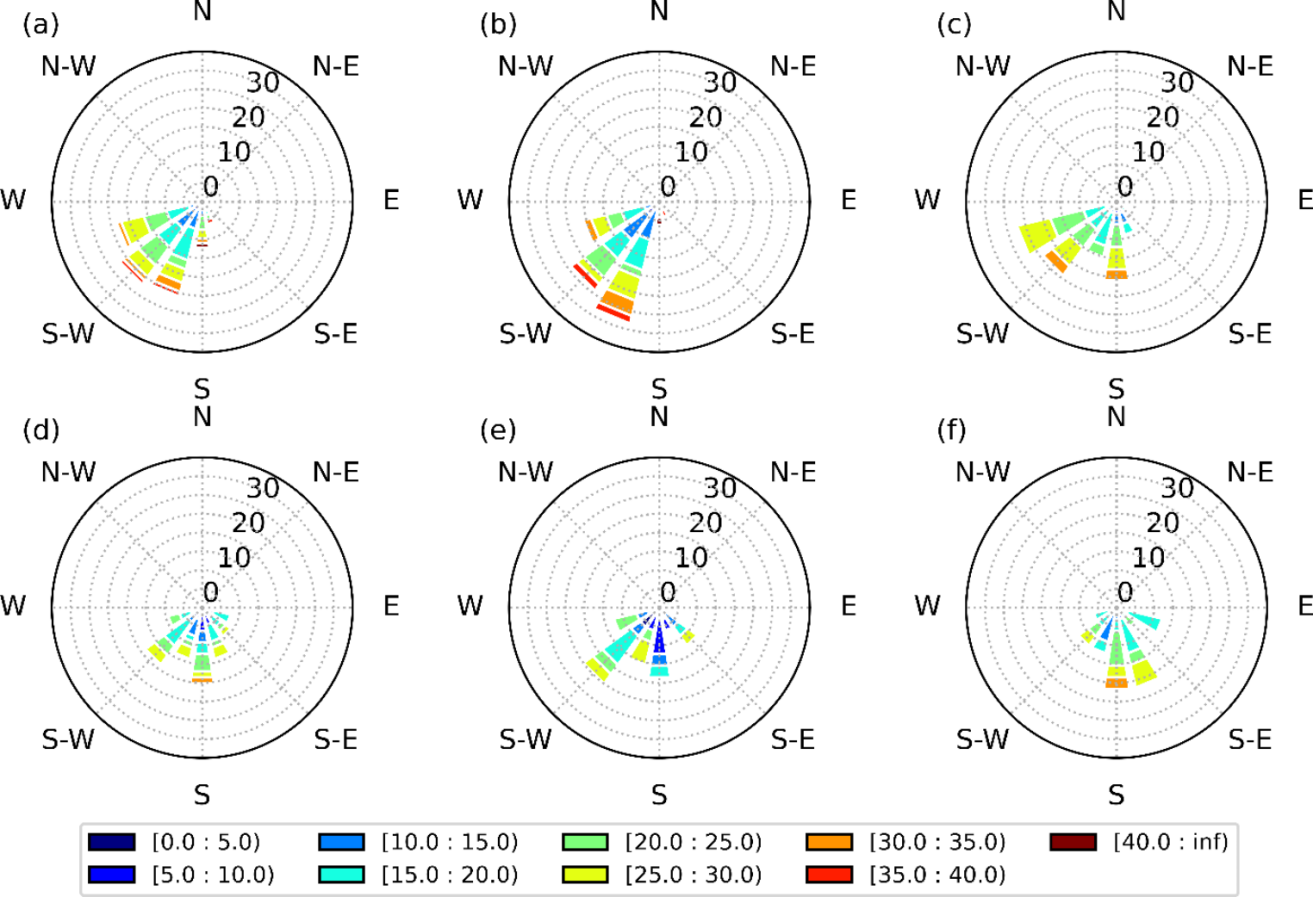
Wind distributions (in %) at 850 hPa for (a)-(c) CTRL and (d)-(f) PGW simulations during (a, d) all, (b, e) SD and (c, f) LD freezing rain events at YUL. The colormap represents the wind speed (m/s) distribution

We also investigated wind speed and direction at 850 hPa, since they contribute to the presence of warm air aloft at YUL (Fig. 13). During freezing rain events, the 850 hPa-wind speeds are generally stronger and blow from the southwest in the CTRL simulation, whereas the PGW produced a broader wind direction distribution, from southwest to southeast. The stronger winds in the current climate at this location occurred during the short-duration events. The mean wind speed during all events decreased by 3.5 m s^− 1^, from 20.3 m s^− 1^ in the CTRL simulation to 16.8 m s^− 1^ in the PGW simulation. While all events are associated with the same mean wind speed in the historical climate (~ 20 m/s), the short-duration events under PGW conditions are associated with a lower wind speed (15 m/s). Conditions at YQB are generally similar to YUL, except some differences in wind speed (not shown). Despite stronger 10-m northeasterly winds being produced in the PGW simulation in the SLRV, weaker 850-hPa winds occur during freezing rain events. This suggests that wind channeling in the SLRV will continue to facilitate freezing rain events in the future.

**Fig. 14:**
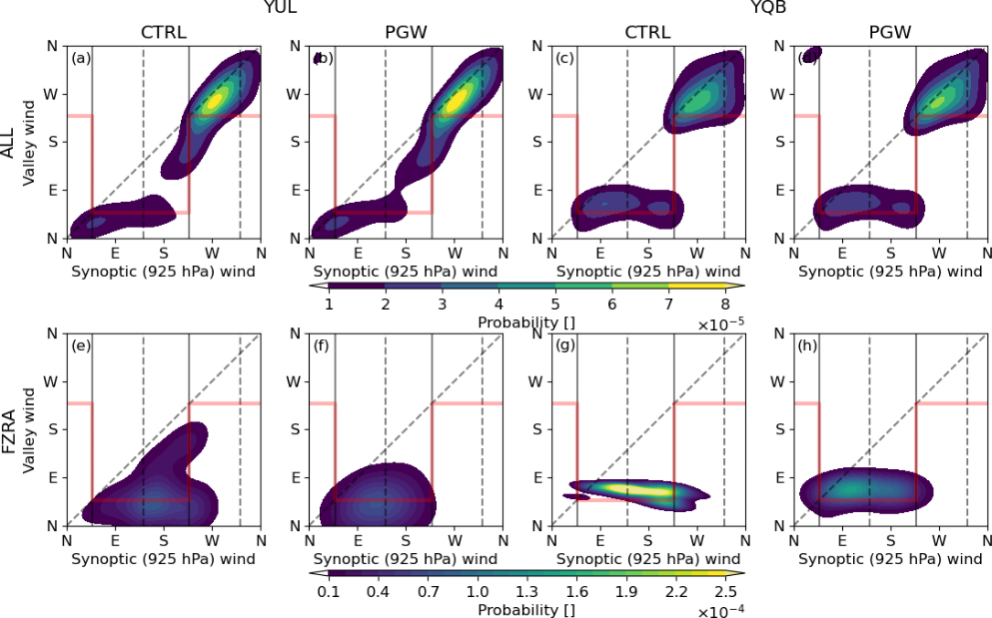
Joint probability distributions of the wind direction within (origin) and above (abscissa) the SLRV for the October–April period (a)–(d) in general and (e)–(h) during freezing rain occurrences at the surface at (a, b, e, f) YUL and (c, d, g, h) YQB for both simulations. The vertical plain and dashed lines represent directions that are parallel and perpendicular to the valley axis, respectively. The red shaded lines are the idealized behavior for pressure-driven channeling

The physical mechanisms that are responsible for wind channeling in the SLRV are identified by studying the distribution of wind directions within and above the valley (Whiteman and Doran 1993; Carrera et al. 2009). Wind direction distributions were generated using a two-dimensional Gaussian kernel density estimation (KDE) with 3-hour wind data at YUL and YQB (Fig. 14). The 10-m winds are used for within-valley distributions and the 925-hPa winds were used for above the valley, because this pressure level is always above the valley walls. Pressure-driven wind channeling occurs when the winds at the surface follow the component of the pressure gradient that is parallel to the valley. This happens when the atmosphere is more stable and can lead to a substantial difference between the wind direction above and within the valley. During freezing rain events, the channeling is mainly pressure-driven, especially at YQB. It is also the main mechanism of wind channeling at YUL. Therefore, the stronger winds at the surface during freezing rain (Fig. 12) are caused by a stronger pressure gradient along the valley axis.

**Fig. 15:**
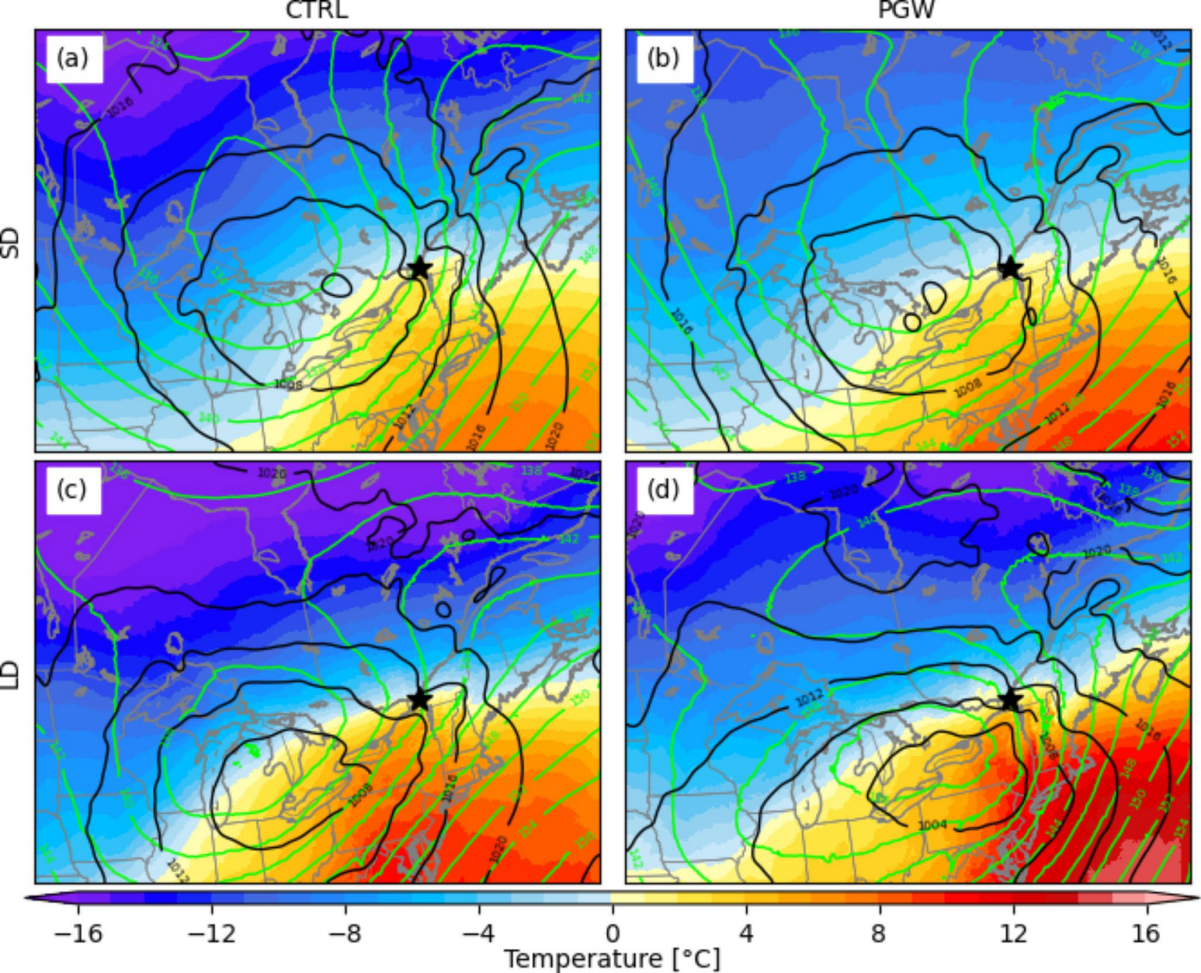
Composite of the SLP (black contours), 850 hPa height (green contours) and temperature (colormap) at the onset time of (a,b) SD and (c,d) LD freezing rain events at YUL (black star)

A composite of sea level pressure (SLP), 850-hPa geopotential height, and 850-hPa temperature at the onset of freezing rain events was produced at YUL in the SLRV (Fig. 15). Only YUL is shown because the composites are similar for YQB. For both simulations, the horizontal temperature gradient is stronger for long-duration events, leading to warmer horizontal air advection. The stronger warm air advection can contribute to maintaining freezing rain for a longer time if weaker cooling processes occur simultaneously. For example, diabatic cooling of melting snow can erode the melting layer aloft (Wexler et al. 1954; Stewart 1985; Kain et al. 2000; Thériault et al. 2012; McCray et al. 2020). During freezing rain events at YUL, the warm air advection is weaker in the PGW simulation for short-duration events, but stronger for longer events. Finally, the strong pressure gradient at the onset of long-duration events enhanced pressure-driven wind channeling.

### Thermodynamic conditions aloft

**Fig. 16:**
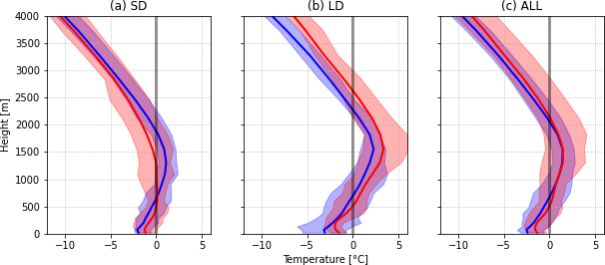
Mean vertical temperature profiles (solid line) at YUL during (a) short-duration (SD), (b) long-duration (LD) and (c) all (ALL) freezing rain events for the CTRL (blue) and PGW (red) simulations. The shaded area is the 25th–75th percentile range

The vertical temperature structures in both simulations were compared in Fig. 16 at YUL. Compared to the current climate, the depth of the melting layer aloft under PGW conditions was on average 196 m thicker during freezing rain events, especially during long-duration ones. The mean thickness of the refreezing layer remained constant in both simulations. Both melting and sub-freezing layers are generally thicker during long-duration events and thinner during short-duration events for both simulations. In the PGW simulation, the melting layer is on average 327 m thicker and the maximum temperature of the melting layer aloft increased by around ~ 1 °C on average during freezing rain events. The mean minimum temperature of the refreezing layer in the PGW simulation is also ~ 1 °C warmer. The 25th–75th percentile spread is larger in the PGW simulation, especially at a higher altitude. Therefore, a wider range of atmospheric conditions will lead to freezing rain occurrence in a warmer climate.

In summary, thicker and warmer melting layers aloft under PGW conditions are consistent with the generally warmer conditions that occur during freezing rain events in the SLRV, in particular at YUL. In warmer conditions, weaker warm air advection could produce the above-0 °C layer aloft, but stronger northeasterly winds within the SLRV would be needed to produce and maintain the refreezing layer near the surface.

### Locations of significant changes

A substantial increase in median annual freezing rain occurrences was observed at Rivière-du-Moulin wind farm (RDM) in warmer climate conditions (Fig. 6a). Since this location is not in the SLRV, the conditions that lead to freezing rain are not linked to wind channeling. The distribution of event duration (Fig. 11c) shows a general increase in the number of freezing rain events under PGW conditions, with three times as many long-duration events (≥ 6 h). The increase in freezing rain occurrence is due to many factors. First, the annual occurrence of precipitation at this location has increased by 15%, from 688 h/y in historical climate to 794 h/y in a warmer climate, but the freezing rain hours increased by 35%, from 26 h/y to 35 h/y in the PGW simulation. Second, freezing rain associated with a supercooled warm rain process increases from 0.9 h/y in the CTRL simulation to 3.7 h/y for the PGW simulation, but a smaller increase in freezing rain produced by the melting solid precipitation of 7.6 h/y occurs (42.2 h/y in the CTRL simulation to 49.8 h/y in the PGW simulation). Therefore, the increase in freezing rain occurrence in a warmer climate at this location is mainly due to an increase in the occurrence of a melting layer aloft combined with an increase of supercooled warm rain occurrences.

**Fig. 17:**
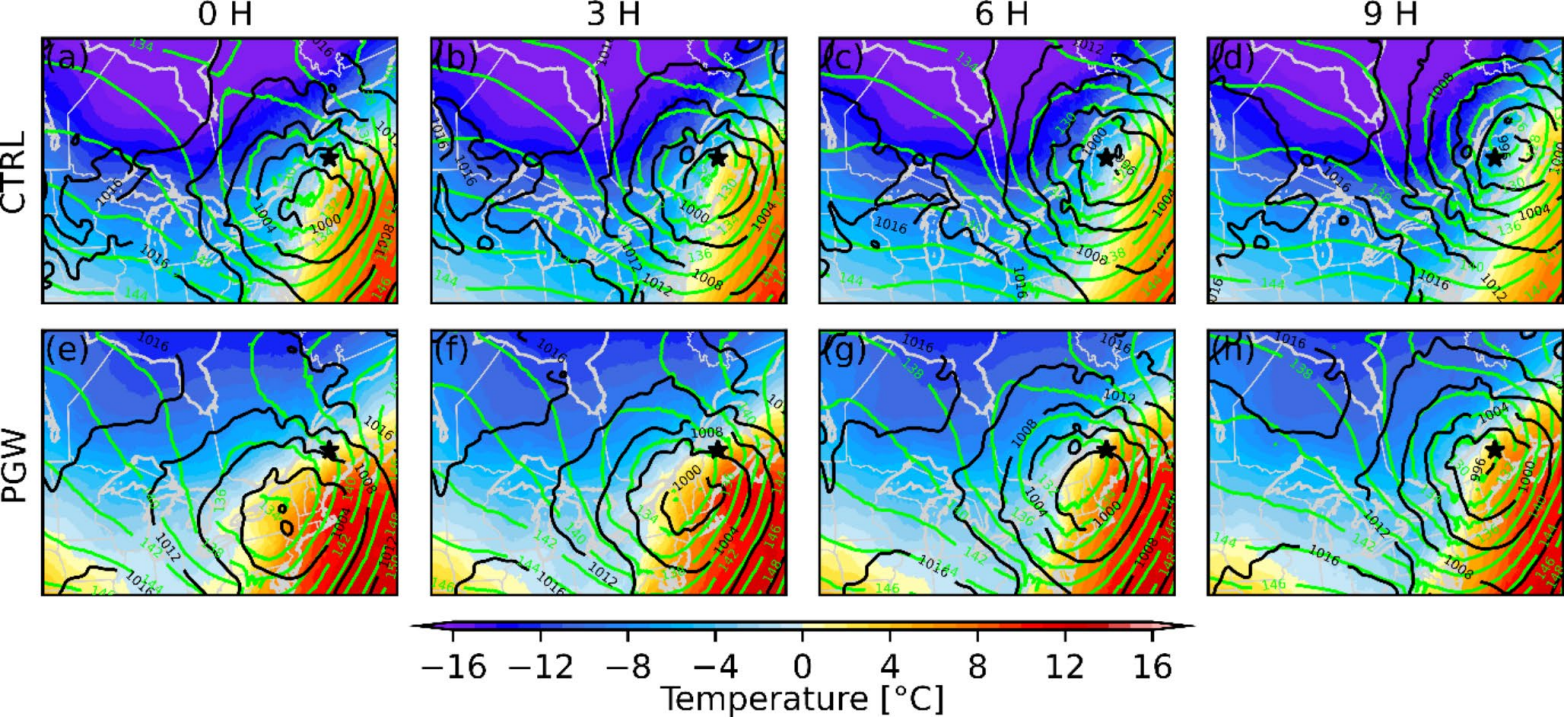
Composite of the SLP (black contours), 850 hPa height (green contours), and temperature (colormap) at 3-hour intervals after the onset of concurrent freezing rain events that were SD in the current climate and LD in the PGW simulation. The star indicates the location at which the freezing rain events were diagnosed (NME)

Even though the median annual freezing rain hours over northern Maine are similar in both CTRL (21 h) and PGW (22 h) simulations, the annual freezing rain hours attributed to long-duration events in the PGW simulation increased (Figs. 7f and 8f). For example, the total freezing rain hours is higher in a warmer climate for events that are longer than 10 h (Fig. 11d). Also, a significant portion of long-duration events in the PGW simulation are linked to similar synoptic conditions but are categorized as short-duration events in the CTRL simulation (Fig. 10b, f). This is due to the delayed onset of freezing rain within the precipitation event. Compared to CTRL, concurrent precipitation events for the PGW approach last up to 2.4 h longer, but freezing rain lasts more than two times longer (5.4 h) during these events. Precipitation events for PGW generally start slightly earlier (1.5 h) and end significantly later (2.8 h), and freezing rain also starts earlier during those events (~ 10.9 h in the CTRL simulation vs. ~ 5.5 h in the PGW simulation). Therefore, these precipitation events have slightly longer durations in the PGW simulation, and freezing rain starts much earlier and lasts way longer in the warmer climate. Figure 17 shows the composite temporal evolution of these concurrent events 9 h after the onset of freezing rain. In the PGW simulation, freezing rain starts earlier than in the CTRL simulation and therefore the location is farther northeast of the center of the low-pressure system. The low-pressure system also moves slightly slower in the PGW simulation. Note that even if PGW does not impact the storm tracks, warmer conditions lead to mesoscale changes in storm location and propagation speed. Finally, 850-hPa warm air is present for a longer duration in the PGW simulation compared to CTRL, once the events commence.

## Summary and conclusions

### Summary

Convection-permitting regional climate simulations for the current climate over most of North America and the Pseudo Global Warming (PGW) method were used to study the climatology of freezing rain events over eastern Canada. First, the median annual hours of freezing rain were computed over the domain and compared with observations from 52 NOAA ISD stations (McCray et al. 2019). The impact of warmer climate conditions on the horizontal distribution of freezing rain occurrences from October to April was investigated by comparing the CTRL and PGW simulations. Despite PGW showing a decrease in the number of freezing rain hours over most of the domain, some areas, such as Bas-Saint-Laurent (BSL), northern Maine (NME) and areas near Québec City (YQB), show an annual increase in freezing rain hours. The increase in freezing rain hours over these areas mainly occurs during the winter (typically in February) and is caused by an increase in the number of long-duration events (≥ 6 h).

Since the PGW method used in these simulations assumes the effects of climate change are mainly thermodynamic, freezing rain events that occur in both the CTRL and PGW simulations within a 12-hour period (concurrent events) were investigated. In the region containing NME and BSL, about half of the freezing rain events are concurrent. Nearly half of these concurrent events were short-duration events in the CTRL simulation that became long-duration events in the PGW simulation. This means that the increase in freezing rain hours (over the areas in the PGW simulation) is partially caused by an increase in the duration of events initiated by similar synoptic systems. This was investigated at a location in NME, in which the conditions favoring freezing rain occur earlier relative to the onset of the precipitation events, the systems move slightly slower in the warmer climate, and air warmer than 0 °C aloft lasts longer during the events.

Within the SLRV, the distribution of freezing rain event durations remained mostly constant in PGW conditions. We found that the pressure-driven component of wind channeling in the SLRV is enhanced by the pressure gradients in PGW conditions during freezing rain episodes. The characteristics of the melting and sub-freezing layers were consistent with the warmer climate changes at this location. Warmer ambient air aloft in PGW conditions means that weaker synoptic winds can sustain the melting layer, while stronger pressure gradients along the valley axis help sustain a sub-freezing layer near the surface.

An increase in the number of events was observed in the PGW simulation, especially for events that last longer than 3 h, at the location north of the SLRV (Rivière-du-Moulin, RDM). The increase in freezing rain occurrence in the warmer climate was explained by three factors: (1) the total precipitation increased by 34%, (2) the frequency of freezing rain formed by supercooled warm rain increased (26.5% of the total increase), and (3) the frequency of a warmer-than-0 °C layer aloft and subfreezing temperatures at the surface increased by 11.6%.

The assessed impacts of a warmer climate are similar to what was found by Matte et al. (2018), with some differences in event duration. The increase in long-duration events in the regions of BSL, NME and YQB were not present in their simulations. This is likely due to the horizontal grid spacing used. They used the fifth-generation Canadian Regional Climate model (CRCM5) with a 0.11° grid mesh (about 12-km grid spacing) for the 1980–2009 and 2070–2099 periods. They did not use a microphysics scheme, since they compared freezing rain diagnostic algorithms. The Sundqvist resolved-scale condensation model (Sundqvist et al. 1989) was used to solve condensation, and both shallow and deep convection schemes were used. Their threshold for an hour of freezing rain was 1 mm/day, which is about 5 times lower than the value used in our study. This 1 mm/day threshold was used by St-Pierre et al. (2019) for their 0.11° grid mesh simulations with the CRCM5 from 1979 to 2014. The pattern of freezing rain occurrence from their research is similar to that of our study, with the exception of the SLRV, where St-Pierre et al. diagnosed more freezing rain. The differences can be explained by the period of simulation, which in their case, included a major ice storm that hit northeastern North America in January 1998. The rare event, which saw precipitation levels reach 100 mm, greatly influenced the climatology of that period (Higuchi et al. 2000; Gyakum and Roebber 2001).

### Conclusions

To conclude, the occurrence of freezing rain shifted northward and increased in February, in warmer climate conditions from a PGW simulation. The increase in freezing rain hours can be partly explained by the longer-lasting melting layer aloft, and by the fact that this layer is present earlier in the start of the precipitation event in the PGW conditions for concurrent freezing rain events over Bas-Saint-Laurent (BSL) and northern Maine (NME), where long-duration events were observed in the warmer climate. At Rivière-du-Moulin (RDM), the large increase in freezing rain hours is associated with a higher frequency of an above-0 °C layer aloft and subfreezing temperatures near the surface, although there is also an increase in freezing rain and drizzle produced by the supercooled warm rain process. Finally, pressure-driven wind channeling within the Saint-Lawrence River Valley is enhanced by slightly steeper pressure gradients during freezing rain events. The 10-m northeasterly winds are stronger and the 850-hPa southerly winds are weaker, making the surface sub-freezing layer last longer and the warm air advection aloft slightly weaker.

In this work, we demonstrated that when thermodynamic changes are mainly considered, the conditions for freezing rain production will change in the future. The spatial distribution of freezing rain occurrences will change depending on local conditions, including wind channeling, the timing of precipitation events, and the characteristics of melting and sub-freezing layers. This will have an impact on the ice accretion on structures, in particular for the areas where freezing rain events will increase in duration. Self-limiting processes produced by the latent heat release from the freezing of supercooled drops at the surface (Lackmann et al. 2002) should be addressed in future studies. The detailed model outputs needed to quantitatively investigate this process further were not available in these simulation datasets. Changes in the precipitation intensity could impact this self-limiting process. Further studies are needed to assess the changes in freezing rain intensity and its impact of near surface warming from the freezing of freezing rain at the surface.

The PGW approach is used to investigate the impact of the thermodynamics feedback in warmer conditions on storms. In contrast, the PGW approach does not account for changes in storm tracks. The pattern of the general circulation will change with global warming (Harvey et al. 2012; Lehmann et al. 2014; Shaw et al. 2016) which will impact the large-scale synoptic conditions leading to freezing rain. A possible poleward shift would lead to a change in the occurrence of freezing rain. High resolution simulations using transient climate change approaches would allow the study of such changes in the future. Nevertheless, PGW simulation allowed us to compare similar synoptic conditions in a current and warmer climate to identify the changes in fine-scale processes leading to freezing rain due to the changes in the thermodynamic conditions.

Overall, this study showed that freezing rain will still occur over eastern Canada in warmer conditions. In addition to the warmer large-scale conditions, fine-scale precipitation processes and topography are also key factors impacting freezing rain events. Further investigation is needed to better understand the processes leading to freezing rain in the future.
